# Acute bilateral visual loss after excessive water intake during a fever

**DOI:** 10.5935/0004-2749.2022-0362

**Published:** 2023-04-10

**Authors:** Hua Wang, Yuanfeng Xue, Bangtao Yao

**Affiliations:** 1 Department of Ophthalmology, Nanjing Lishui District Baima Health Hospital, Nanjing, Jiangsu Province, China; 2 Department of General Practice, Nanjing Lishui People’s Hospital, Zhongda Hospital Lishui branch, Southeast University, Nanjing, Jiangsu Province, China; 3 Department of Ophthalmology, Nanjing Lishui People’s Hospital, Zhongda Hospital Lishui branch, Southeast University Nanjing, Jiangsu Province, China

Dear Editor,

Glaucoma is proven to be the leading cause of irreversible visual impairment, affecting 67 million human population worldwide^(1,2)^. Approximately 9%-12% of older people (aged >65 years) develop glaucoma in the United Kingdom^(3)^.

Acute angle-closure glaucoma is an urgent ophthalmic disorder caused by the sudden obstruction of the aqueous drainage pathway and is usually unilateral^(1)^. Pupillary block angle-closure glaucoma mainly occurs in patients with increased lens thickness, hypermetropic, or shallow anterior chamber^(4,5)^. Acute angle-closure glaucoma is diagnosed based on the painful sudden blurring of vision, elevated intraocular pressure, and shallow/closed anterior chamber^(6)^.

**Figure 1:**
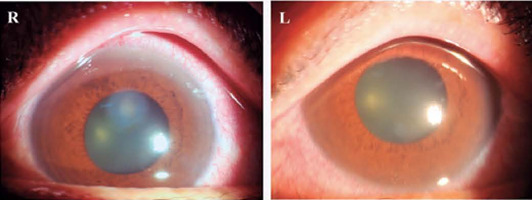
On slit-lamp examination, conjunctival congestion, diffuse corneal edema, and mid-dilated non-reacting pupils were noted in both eyes.

A woman in her late 50s presented to our emergency ophthalmology department with a 4-h history of painful sudden reduction of vision in both eyes, in addition to nausea and headache. Detailed history revealed that the patient had a fever for 1 day after taking the advice of the physician to drink lots of water. She drank 5 L of water. Her systemic histories were negative. On admission, her temperature was 36.9°C, and the best-corrected visual acuities were 20/300 and 20/160 in the right and left eyes, respectively. On slit-lamp examination, conjunctival congestion, diffuse corneal edema, and mid-dilated fixed pupils were noted in both eyes (Figure 1). The anterior-segment spectral-domain optical coherence tomography showed shallow anterior chambers in both eyes. The intraocular pressures were 57 and 52 mmHg in the right and left eyes, respectively. Gastroenterology and neurology consultations were conducted. All other investigations including computed tomography, routine blood and urine tests, hypersensitive C-reactive protein, erythrocyte sedimentation rate, electrocardiogram, kidney function, electrolyte, and neurological examinations were unremarkable. Based on the above findings, she was diagnosed with bilateral acute angle-closure glaucoma.

**Figure 2:**
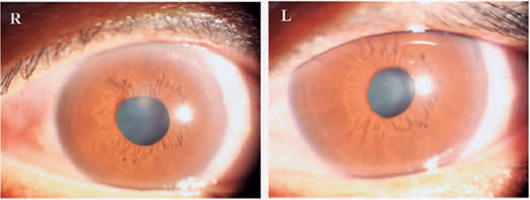
At a 1-day follow-up, her symptoms had resolved significantly.

The patient was treated intravenously with mannitol 20% once, topical pilocarpine 1% four times daily, and topical carteolol 2% twice daily. At a 1-day follow-up, her symptoms had resolved considerably (Figure 2), and her intraocular pressures were 11 and 10 mmHg in the right and left eyes, respectively. The best-corrected visual acuities were 20/30 and 20/20 in the right and left eyes, with a refractive status of +2.75/+2.25 × 100 and +1.75/+0.75 × 140, respectively. The retinal nerve fiber layer thickness was normal. Topical treatment was maintained for 3 days, and the patient had no recurrence of symptoms. She was advised to undergo laser iridotomy.

The water-drinking test has potential significance for primary angle-closure suspects. Choroidal expansion after excessive water intake can result in the anterior displacement of the lens-iris diaphragm and secondary anterior chamber angle obstruction^(5)^. Therefore, excessive water intake by patients with fever may be a risk factor for acute angle-closure glaucoma.

Acute angle-closure glaucoma may be easily misdiagnosed as acute gastroenteritis, increased intracranial pressure, meningitis, or electric ophthalmia because patients often present with headache, nausea, vomiting, and painful red eye^(4,7)^.

Urgent treatment is required to protect the visual function^(6)^. Lowering intraocular pressure is the primary principle in the treatment of acute angle-closure glaucoma. Evidence shows that the reduction of intraocular pressure significantly slowed down the progression to optic nerve damage^(3)^. Topical intraocular pressurereducing drugs (e.g., pilocarpine, brinzolamide, and carteolol) were effective for patients with angle-closure glaucoma. Intravenous administration of acetazolamide and mannitol was also recommended ^(3,8)^.

Prophylactic laser iridotomy should be performed in both eyes with pupillary block angle-closure glaucoma, immediately following the lowering of the intraocular pressure^(4)^. Surgical methods (e.g., trabeculectomy and lens extraction) are appropriate options for glaucoma, particularly if medical or laser treatments failed^(4,9)^.
